# Results from the First-in-Human Study of the Caterpillar™ Arterial Embolization System

**DOI:** 10.1007/s00270-022-03300-1

**Published:** 2022-11-30

**Authors:** Andrew Holden, Bibombe P. Mwipatayi, Manar Khashram, Steven Dubenec, Gerard S. Goh, Richard A. Settlage

**Affiliations:** 1 Auckland City Hospital, School of Medicine, University of Auckland, 2 Park Road, Grafton, Auckland, 1023 New Zealand; 2 Royal Perth Hospital, University of Western Australia, Perth, Australia; 3 Waikato Hospital, University of Auckland, Hamilton, Waikato New Zealand; 4 Vascular Associates, Royal Prince Alfred Hospital, Sydney, Australia; 5 Departmemt of Surgery, Central Clinical School, Monash University, Alfred Health, Melbourne, Australia; 6 Becton, Dickinson and Company, Tempe, AZ USA

**Keywords:** Embolotherapy, Percutaneous transcatheter embolization, Peripheral artery embolization

## Abstract

**Purpose:**

To assess occlusion success and adverse events associated with the use of a self-expanding device for peripheral artery embolization.

**Methods:**

This prospective, single-arm, feasibility study was conducted using the Caterpillar™ Arterial Embolization Device composed of opposing nitinol fibers and a flow-occluding membrane. Twenty patients (24 embolization sites) were treated at four investigational centers in New Zealand and Australia and followed for 30 days. Embolization sites included mesenteric, accessory renal, and iliac arteries and their branches. Primary outcome measures were peri-procedural occlusion confirmed by angiography and freedom from device-related serious adverse events (SAEs) at 30 days. Secondary observations included time to occlusion and assessment of adverse events.

**Results:**

Peri-procedural occlusion was 100%, and freedom from a device-related SAE was 94.7% at 30 days. One patient had abdominal bloating that required hospitalization deemed possibly related to the device or procedure. Twenty-two of 24 embolization sites were occluded with one device (91.7%). Mean procedure duration was 11.7 ± 8.6 min (device deployment time: 1.8 ± 1.0 min), and mean fluoroscopy time was 241 ± 290.7 s. All embolization sites occluded during the procedure with 62.5% occluded within three minutes and 91.6% occluded within ten minutes. No devices migrated or required re-embolization. Freedom from device- and procedure-related adverse events was 84.2%. One patient died from aortic rupture during a subsequent adjunctive abdominal aortic endovascular procedure deemed unrelated to the embolization device or procedure.

**Conclusions:**

This first-in-human study of the Caterpillar embolization device achieved peri-procedural occlusion in all patients with a 94.7% freedom from device-related SAE at 30 days.

**Level of Evidence:**

Level 2b—prospective, multicenter, single-arm, first-in-human clinical study. Pre-specified endpoints were analyzed using descriptive statistics.

## Introduction

With the number of applications for peripheral vascular embolization and the wide range of vessel sizes to be treated, there is no single agent or device that fits all indications [1–4]. Mechanical embolization devices such as pushable or detachable coils have been a standard of care for decades and come in various shapes, sizes, metallic types (e.g., stainless steel and platinum), and degrees of thrombogenicity (e.g., Dacron or nylon fibers). Coils, however, can be difficult to position and control, can be prone to migration, and multiple coils are generally required to achieve complete occlusion. Embolization plugs, such as the Amplatzer vascular plug (Abbott), the MVP microvascular plug (Medtronic), the AZUR vascular plug (Terumo), and IMPEDE embolization plug (Shape Memory Medical), are designed with thrombogenic metal fibers and sometimes with occlusion membranes. They offer the possibility of controlled deployment with limited migration, and vessels can often be occluded with one plug minimizing the need for multiple devices. Since the use of multiple devices increases fluoroscopy exposure to patient and operator, overall procedure time and cost, and the risk of complications, the ability to achieve occlusion with as few devices as possible is desirable [1, 5–7].

The Caterpillar arterial embolization device used in this prospective study is a plug composed of opposing nitinol self-expanding fibers to promote thrombosis with a proximal membrane to provide rapid occlusion. It was designed for placement in a wide range of arteries with the objective of minimizing the number of devices needed to achieve occlusion. This first-in-human trial was designed to assess occlusion success and adverse events associated with the use of the device.

## Methods and Materials

### Study Design

Twenty patients were treated in the prospective, multicenter, single-arm study entitled, Clinical Use of the Caterpillar™ Arterial Embolization Device System for Arterial Embolization in the Peripheral Vasculature (CHRYSALIS). Investigators at five centers in New Zealand and Australia enrolled patients between September 2019 and September 2020 under a protocol approved by their institutional ethics committees. Patients provided written informed consent prior to participation in the study, and procedures were conducted in accordance with the Declaration of Helsinki, good clinical practices, and applicable healthcare laws in both countries. Data were collected by the investigators using standardized web-based clinical report forms. An independent medical monitor reviewed all complications for adverse event (AE) trends. The study was sponsored by C.R. Bard/Becton, Dickinson and Company and was registered on clinicaltrials.gov [NCT04090320] prior to patient enrollment.

### Study Device

**Fig. 1:**
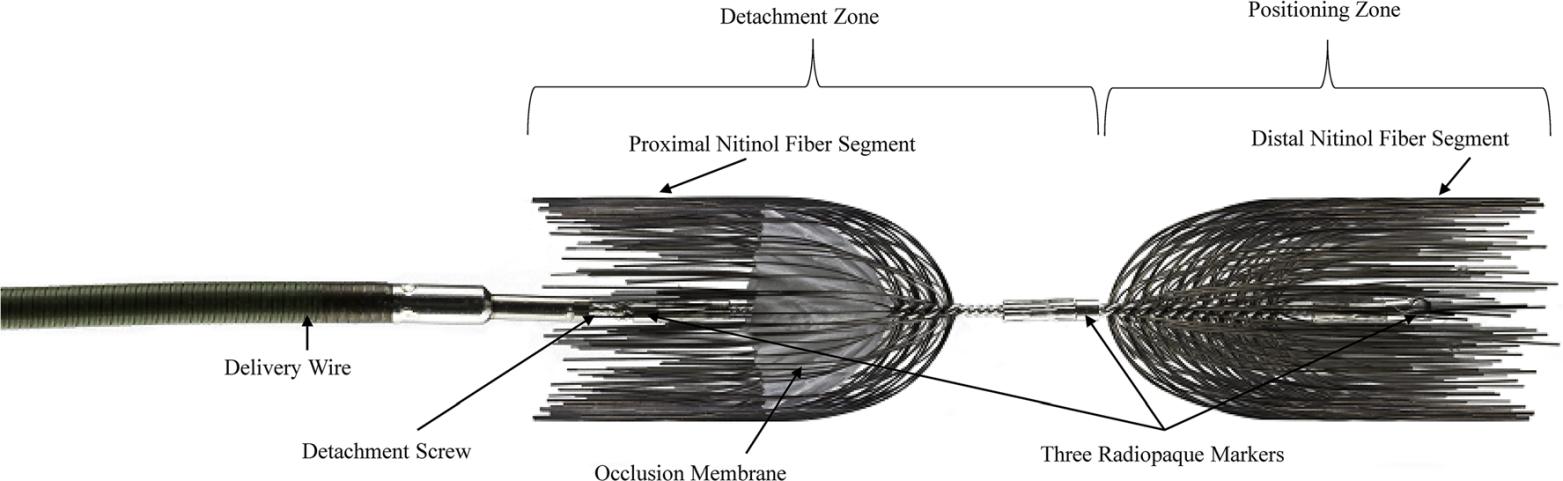
Caterpillar™ and Caterpillar™ Micro Device Components (Becton, Dickinson and Company, Tempe, AZ, USA). The self-expanding arterial embolization devices were designed for peripheral arterial embolization. Devices were composed of a cobalt-chromium stem, nickel-titanium fibers, a polyurethane and polyethylene occlusion membrane, and platinum-iridium radiopaque marker bands. There were two Caterpillar device sizes (038 and 056) for 3–7-mm-diameter arteries (device landing zone: 26–37 mm) and one Caterpillar Micro device (027) for 1.5–4-mm-diameter arteries (device landing zone of ≥ 16 mm)

Patients were treated with the Caterpillar™ or Caterpillar™ Micro Arterial Embolization Device (Becton, Dickinson and Company, Tempe, AZ, USA), self-expanding vascular occlusion devices intended as permanent implants for peripheral arterial embolization. The devices, described briefly in two previous publications and depicted in Fig. 1, are composed of a cobalt-chromium stem, opposing nickel-titanium (i.e., nitinol) fibers, a polyurethane and polyethylene occlusion membrane, and platinum-iridium radiopaque marker bands [8, 9]. When deployed, the nitinol fibers form an opposing concave, hour-glass shape designed to provide stability inside the artery and encourage thrombosis while the occlusion membrane lining the inside of the proximal fibers is designed to block blood flow and support rapid occlusion. Caterpillar devices are packaged sterile and intended for single use, and the system includes the implant, loader, dispenser hoop, torque tool, and detachable delivery wire. There are two Caterpillar device sizes (038 and 056) available for arteries with a target diameter of 3 to 7 mm and a landing zone of 26 to 37 mm and one Caterpillar Micro device (027) designed for arteries with a target diameter of 1.5 to 4 mm and a landing zone of ≥ 16 mm (Table 1). The device sizes of 027, 038, and 056 refer to the inner diameter compatibility of the delivery catheter in inches (i.e., 0.027″, 0.038″, and 0.056″).

**Table 1:** Device sizing and compatible catheters

Product name	Product reference	Target artery diameter (mm)	Marker to marker length^1^ (mm)	Maximum deployed length^2^ (mm)	Delivery wire length (cm)	Delivery catheter compatibility: inner diameter (in/mm)	Compatible catheters (Outer diameter French size)^3^
Caterpillar™ Micro	027	1.5–4	7	16	170	0.027 / 0.686	Terumo Progreat™ (2.8F/3.0F)
							Boston Scientific Renegade™ Hi-Flo (2.8F/3.0F)
							Boston Scientific Direxion™ Hi-Flo (2.8F/3.0F)
							Merit EmboCath™ Plus (2.9F/3.0F)
							Stryker Excelsior™ (2.7F/2.9F)
							Codman Prowler™ (2.6F/3.0F)
							Medtronic Marksman™ (2.8F/3.2F)
							Medtronic Rebar™ (2.8F)
							Cook Cantata™ (2.9F)
Caterpillar™	038	3–6	17	26	155	0.038 / 0.965	Boston Scientific Imager™ II (5F)
							Cook Beacon™ Tip (5F)
							Cordis Tempo™ (4F or 5F)
	056	5–7	18	37	155	0.056 / 1.422	Cordis Vista Brite Tip™ (5F)
							Merit Concierge™ (5F)
							Codman Envoy™ (5F)

^1^Marker to Marker Length: Distance between the distal and proximal radiopaque markers

^2^Maximum Deployed Length: Length from the distal fiber tips to the proximal fiber tips in the minimum target artery diameter

^3^Compatible Catheters: Caterpillar Arterial Embolization devices have been tested for compatibility with the listed delivery catheters. The delivery catheter length must be less than or equal to 140 cm (155 cm for the Micro Device)

### Study Eligibility

Patient inclusion and exclusion criteria are detailed in Table 2. Eligible patients had a peripheral arterial embolization site or multiple sites that could be treated with an appropriately sized study device. Angiography was required to demonstrate that the embolization site was in an artery that could accommodate the device diameters and lengths. Patients were excluded if the embolization site was in a vein; in a head, neck, heart, or coronary artery; crossed highly locomotive joints or muscle beds (e.g., elbow, hip, knee, shoulder, thoracic inlet/outlet); or was in a high-flow vessel with a significant risk of migration (e.g., pulmonary arteriovenous malformations). Patients receiving anticoagulation or antiplatelet therapy that in the opinion of the investigator would clinically interfere with study outcomes were also excluded (e.g., direct thrombin inhibitors, factor Xa inhibitors, and vitamin K antagonists).

**Table 2:** Inclusion and exclusion criteria

*Inclusion criteria*
Clinical inclusion criteria
1. Signed and dated Informed Consent Form (ICF) before data collection or performance of study procedures 2. Male or non-pregnant female ≥ 18-years-old willing and able to comply with protocol requirements, and with a life expectancy sufficient to allow for completion of the study 3. Peripheral artery target embolization site(s) that could be treated with the Caterpillar Arterial Embolization Device according to the Instructions for Use (Note: More than one site could be treated per patient)
Angiographic inclusion criteria
4. Embolization site in a native artery: a. Vessel diameter: 1.5–7 mm (visual estimate) b. Landing zone: ≥ 16 mm (device implant lengths: 16–37 mm) *Exclusion criteria*
Clinical exclusion criteri*a*
1. Access vessel precluded safe insertion of the delivery catheter 2. Embolization site was in a vein, in the head, neck, heart, or coronary vessels, crossed highly locomotive joints or muscle beds (e.g., elbow, hip, knee, shoulder, thoracic inlet/outlet), or was located in a high-flow vessel where there was significant risk of migration and unintended (non-target site) occlusion (e.g., such as pulmonary arteriovenous malformations) 3. Patient had a known allergy or hypersensitivity to contrast media or to any of the device materials including cobalt, chromium, nickel, titanium, platinum, iridium, polyurethane, or polyethylene that could not be adequately pre-medicated 4. Patient had a known uncontrolled blood coagulation or bleeding disorder, or was to receive anticoagulant or antiplatelet therapy before, during, or after treatment that could have interfered with the study endpoints (e.g., direct thrombin inhibitors, factor Xa inhibitors, and vitamin K antagonists) 5. Patient had an unresolved systemic infection, abnormal results from a preoperative laboratory test or physical examination, had a connective tissue disorder (e.g., Ehlers–Danlos syndrome), arteritis (e.g., Takayasu’s disease), or another circulatory disorder that could have interfered with the study endpoints 6. Patient had another medical condition that may have caused noncompliance with the protocol, confounded data interpretation, or shortened life expectancy enough to prevent completion of study procedures and follow-up 7. Patient was participating in an investigational drug or another device study that had not completed study treatment or interfered with the study endpoints (Note: Studies requiring extended follow-up visits for products that had subsequently become commercially available were not considered investigational studies)

### Outcome Measures

The primary outcome measure was technical success defined as peri-procedural occlusion of the embolization site confirmed by the investigator using angiography. The primary safety measure was 30-day freedom from device-related serious adverse events (SAEs) defined as adverse events caused by the embolization device that led to death or serious deterioration in health (e.g., prolonged hospitalization, permanent impairment, or life-threatening injury or illness). Secondary measures included time to occlusion reported as the percentage of embolization sites occluded during the first ten minutes after deployment (angiographic visualization by the investigator at one-minute intervals); freedom from clinically-relevant recanalization through 30 days defined as freedom from blood flow through the treatment site that required reintervention; freedom from clinically-relevant migration of the embolization device requiring reintervention through 30 days, and freedom from device- or procedure-related adverse events (AEs) through 30 days. In addition, investigators answered three questions based on their use of the device during the procedure: (1) Was the device easily tracked and delivered to the target embolization site? (2) Was fluoroscopic visibility of the device acceptable? (3) Was the device accurately deployed at the target site? All answers were qualitative based on the investigator’s opinion and answered “yes” or “no”.

### Study Procedures and Follow Up

Patients were enrolled in the study after providing written informed consent and receiving a comprehensive physical examination, preoperative laboratory tests, and angiographic assessment of eligibility. Once an appropriately-sized device was chosen based on vessel anatomy and dimensions, the device loader was advanced through a Tuohy–Borst adapter until seated against the delivery catheter hub. The Caterpillar device was advanced from the loader through the delivery catheter to the catheter tip using the attached delivery wire. Patients were considered treated when the embolization device exited the system loader and entered the delivery catheter. The delivery catheter was positioned just distal to the embolization site, the device was held stationary using the delivery wire, the delivery catheter was retracted to deploy the device, and the delivery wire was turned counterclockwise using the supplied torque tool to separate it from the deployed device. The Caterpillar device could be recaptured and repositioned after deployment of the distal positioning zone; if, however, device position was incorrect after the detachment zone was deployed, both the device and delivery catheter had to be recaptured by the guiding sheath, removed together, and discarded. The Caterpillar Micro device could not be recaptured in the microcatheter and repositioned once the distal fiber segment was deployed.

A single device per embolization site was recommended in the protocol; however, additional devices could be deployed to achieve occlusion if required. The protocol recommended that unless a delay impacted patient safety, the investigator should wait at least five minutes before deploying another embolization device or agent so that the original study treatment could be evaluated. Medications and adjunctive medical therapy before, during, and after the study procedure were administered per the investigator’s standard of care and documented through completion of the study. Adjunctive embolization devices or agents were allowed to treat the target site if deemed clinically necessary and were documented accordingly. An in-office or telephone follow-up visit was performed at 30 (−7/ + 21) days, and all available medical records and embolization site images were reviewed and documented.

### Statistical Analysis

CHRYSALIS was designed as an exploratory study. The sample size was based on the potential adequacy of data to meet the study objectives, not statistical considerations. The analysis population consisted of all patients treated with the study device. There were no statistically-tested hypotheses. Data collected were observational and were summarized using descriptive statistics including frequency counts and percentages for categorical variables and means and standard deviations for continuous variables.

## Results

### Baseline Patient and Lesion Characteristics

**Fig. 2:**
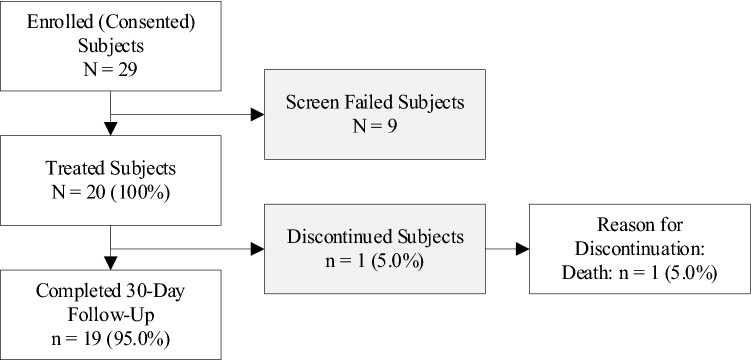
Subject Disposition. Twenty-nine patients were consented and enrolled at five study sites. Nine patients failed to meet the eligibility criteria for inclusion in the trial, while 20 patients were treated with the Caterpillar embolization device at four of the five study sites. Nineteen patients completed the study. Note: One patient had successful embolization of the inferior mesenteric artery completed with an 056 Caterpillar device. During an abdominal aortic endograft procedure the following day, the patient’s aortic aneurysm ruptured resulting in death. The death was deemed by the investigator as unrelated to the embolization device or procedure

**Fig. 3:**
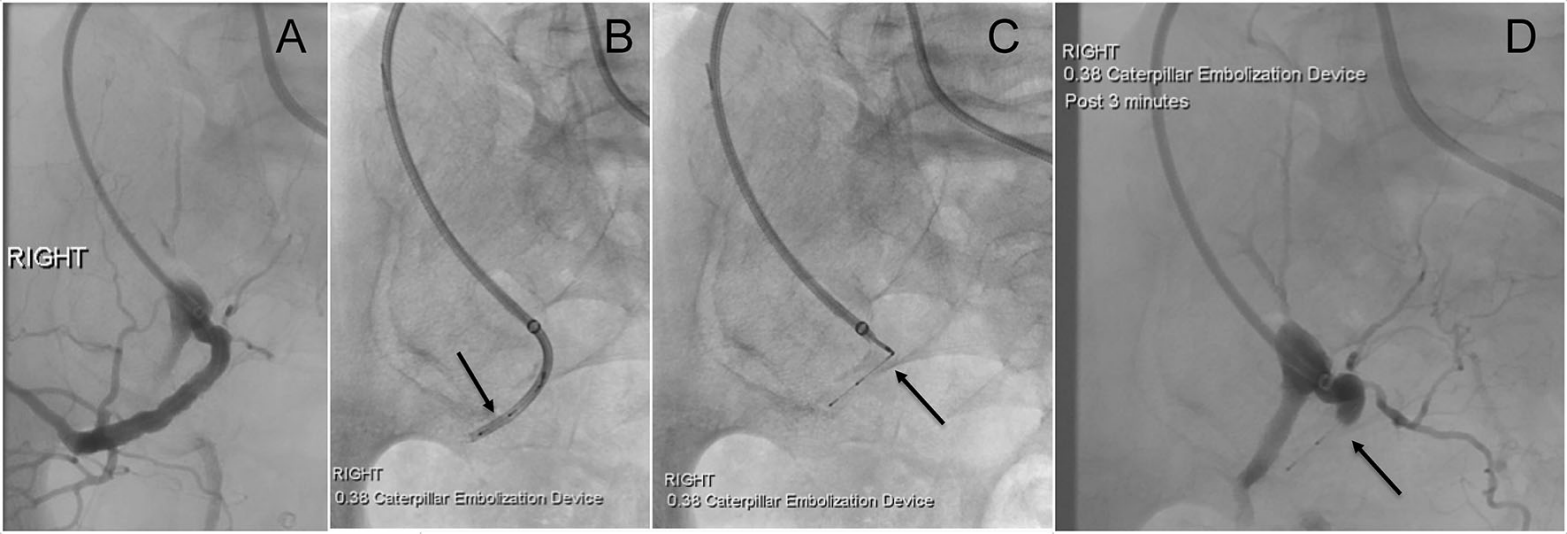
Embolization of the posterior division of the right internal iliac artery prior to EVAR. **A** 6F sheath positioned at the origin of the posterior division of the right internal iliac artery via a contralateral groin approach. **B** Coaxial 5F catheter positioned through the sheath into the proximal target artery with a Caterpillar 038 device introduced and advanced to the catheter tip (*arrow*). **C** Caterpillar 038 device deployed by withdrawing the catheter. Note the detachable delivery wire is still attached at this stage (*arrow*). **D** Angiogram performed 3 min after deployment with complete occlusion of the target artery (*arrow*)

**Fig. 4:**
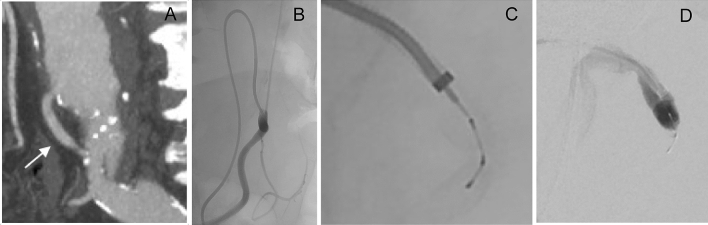
Embolization of the inferior mesenteric artery (IMA) during an EVAR procedure. **A** Large IMA (arrow) arising from an abdominal aortic aneurysm. **B** IMA catheterized during EVAR procedure. **C** Caterpillar 038 device deployed through a 5F catheter into the IMA. **D** The IMA is completely occluded 5 min after deployment

Twenty-nine patients were consented and enrolled at five sites while 20 patients were treated at four of the sites; nine patients failed to meet the eligibility criteria for inclusion in the trial (Fig. 2). Baseline patient demographics and medical histories are summarized in Table 3. Patients were on average 73.4 ± 8.5 years old, and 85% were male. Risk factors included hypertension (90%), past or present smoking (85%), and dyslipidemia (70%) while co-morbidities included aortic disease (60%), peripheral arterial disease (60%), and coronary artery disease (45%). Embolization site characteristics and procedural details are summarized in Table 4. Twenty-four peripheral arterial embolization sites were treated across seven arterial locations with the most common areas of treatment in accessory renal arteries, internal iliac arteries, iliac artery branches (Fig. 3), and inferior mesenteric arteries (Fig. 4). The mean treated artery diameter was 4.5 ± 1.2 mm with an average landing-zone length of 44.5 ± 12.7 mm. Sixteen patients (80%) had one site treated while four patients (20%) had two sites treated.

**Table 3:** Baseline patient demographics and medical history

Patient demographics	Treated population (*N* = 20)
Age (years), ${\bar{x}} \,$± SD	73.4 ± 8.5
*Gender, % (n)*	
Male/Female	85 (17)/15 (3)
BMI, kg/m^2^ ± SD	30.8 ± 4.7
*Race, % (n)*	
European	80 (16)
Native New Zealander	5 (1)
Native Australian	5 (1)
Middle Eastern	5 (1)
Asian (Chinese)	5 (1)
*Medical history, % (n)*	
Hypertension	90 (18)
Cigarette smoking	85 (17)
Dyslipidemia	70 (14)
Peripheral arterial disease	60 (12)
Aortic disease	60 (12)
Coronary artery disease	45 (9)
Gastrointestinal disease	30 (6)
Respiratory disease	30 (6)
Chronic kidney disease	20 (4)
Transient ischemic attack (TIA)	15 (3)
Congestive heart failure	10 (2)
Hepatitis	10 (2)
Dialysis	10 (2)
Kidney cancer	5 (1)
Other cancer (non-kidney/liver)	5 (1)
Diabetes mellitus	5 (1)
Stroke	5 (1)

**Table 4:** Embolization site characteristics and procedure details

Embolization site characteristics	Treated population (*N* = 20)
*Embolization sites treated, n*	24^1^
1 Site, % (n/N)	80 (16/20)
2 Sites, % (n/N)	20 (4/20)
*Location, % (n)*	
Inferior mesenteric artery	16.6 (4)
Accessory renal artery	12.5 (3)
Right accessory renal artery	12.5 (3)
Right internal iliac artery	12.5 (3)
Internal iliac branch	8.3 (2)
Right internal iliac side branch	8.3 (2)
Left internal iliac artery	4.2 (1)
Left internal iliac branch	4.2 (1)
Left lumbar artery	4.2 (1)
Proximal superior mesenteric artery	4.2 (1)
Right common iliac artery anterior branch	4.2 (1)
Right common iliac artery posterior branch	4.2 (1)
Superior gluteal artery	4.2 (1)
*Reason for treatment, % (n)*	
Prophylactic embolization^2^	95.8 (23)
Type II Endoleak (Post-EVAR/TEVAR)	4.2 (1)
*Treatment site dimension, mm* ± *SD*	
Artery diameter	4.5 ± 1.2
Artery length	44.5 ± 12.7
*Procedural Details*	
Femoral artery access, % (n)	100 (20)
*Device deployment time*,^3^ *min ± SD*	
Mean ± SD	1.8 ± 1.0
Median	2.0
*Procedure duration,*^4^* min* ± *SD*	
Mean ± SD	11.7 ± 8.6
Median	9.0
*Mean fluoroscopy exposure, sec* ± *SD*	
Mean ± SD	241.0 ± 290.7
Median	121.0
*Number of devices to achieve occlusion. n*	26^5^
1 Device, % (n/embolization site)	91.7 (22/24)
2 Devices, % (n/embolization site)	8.3 (2/24)
*Study Device Size, % (n)*^6^	
027 Caterpillar Micro	10.7 (3)
038 Caterpillar	53.6 (15)
056 Caterpillar	35.7 (10)
Device needed to be recaptured, % (n)	0 (0)
Adjunctive embolization devices/agents, % (n)	0 (0)

^1^ 24 embolization sites were treated in 20 patients

^2^ Prophylactic embolization for flow obstruction in preparation for Endovascular Aneurysm Repair (EVAR)

^3^ Device deployment time: Time from device introduction into the delivery catheter until device detachment

^4^ Procedure duration: Time from first study device connection to the sheath to the final embolization site occlusion

^5^ 26 devices were used at 24 embolization sites (20 patients)

^6^ 28 devices were introduced into the delivery system

### Post-Procedure Follow-up and Primary Outcomes

Primary and secondary outcomes are summarized in Table 5. Nineteen patients (95%) completed the study (i.e., one patient died during a subsequent EVAR procedure). The primary outcome measure, technical success or successful occlusion of the target embolization site confirmed by angiography during the procedure, was 100% (24/24 sites). All but two embolization sites (91.7%) were occluded with one device; an accessory renal artery was treated with two Caterpillar 038 devices and an internal iliac artery had a Caterpillar 056 device and 038 device deployed to achieve full occlusion. The primary safety outcome, the proportion of patients free from device-related SAEs through 30 days, was 94.7% (18/19 patients).

**Table 5:** Primary and secondary outcomes

	%, (n/N)	(CI%)
*Primary outcomes*		
Technical success^1^	100 (24/24^2^)	(85.8, 100)
30-Day freedom from device-related SAEs^3^	94.7 (18/19^4^)	(74.0, 99.9)
*Secondary outcomes*		
Time to Occlusion, % (n/N)		
≤ 1 min	16.7 (4/24)	(4.7, 37.4)^5^
≤ 2 min	20.8 (5/24)	(7.1, 42.2)
≤ 3 min	25.0 (6/24)	(9.8, 46.7)
≤ 4 min	0.0 (0/24)	(0.0, 14.2)
≤ 5 min	8.3 (2/24)	(1.0, 27.0)
≤ 10 min	20.8 (5/24)	(7.1, 42.2)
> 10 min	8.3 (2/24)	(1.0, 27.0)
Freedom from recanalization through 30 days	100 (23/23^6^)	(85.2, 100)
Freedom from clinically-relevant acute migration^7^	100 (26/26^8^)	(86.8, 100)
30-Day freedom from clinically-relevant migration	100 (25/25)	(86.3, 100)
30-Day freedom from device- or procedure-related AEs	84.2 (16/19)	(60.4, 96.6)
Qualitative assessments of the embolization device		
Accuracy of delivery	100 (26/26)	(86.8, 100)
Ease of trackability/deliverability	100 (26/26)	(86.8, 100)
Visibility under fluoroscopy	96.4 (27/28^9^)	(81.7, 99.9)

^1^ Successful occlusion of the embolization site as confirmed by the investigator via angiographic assessment during the index procedure

^2^ There were 24 embolization sites treated in the study

^3^ The proportion of patients free from device-related serious adverse events (SAE) through 30-day

^4^ Twenty patients were treated in the study. One patient died prior to the 30-day exam

^5^ Two-sided 95% confidence interval is calculated using the Clopper-Pearson method

^6^ One patient died prior to the 30-Day follow-up, not related to the device or procedure, without having a recanalization event. The embolization site was not evaluable at 30 days and was not included in the analysis

^7^ Clinically-relevant migration: migration of the study device from the target embolization site that required a reintervention

^8^ There were 26 devices used to treat the 24 embolization sites in 20 patients

^9^ 28 devices included all devices inserted into the delivery catheter. Of the devices introduced, one device was reported as difficult to visualize under fluoroscopy

### Secondary Outcomes

The mean device deployment time, defined as the time from device introduction into the delivery catheter until detachment, was 1.8 ± 1.0 min. The mean overall procedure duration was 11.7 ± 8.6 min, and the mean fluoroscopy exposure time was 241.0 ± 290.7 s. No arteries required adjunctive embolization with other devices or embolic agents, no sites required re-embolization, and no devices migrated from the target site requiring a reintervention through 30 days. Time to occlusion was tracked angiographically by the investigator at one-minute intervals during the embolization procedure; 62.5% of the 24 treated sites occluded within 3 min, 70.8% occluded within 5 min, and 91.6% occluded within 10 min of device deployment. Two sites took more than ten minutes to occlude, but full occlusion was achieved before the end of the embolization procedure. A total of 84.2% (95% CI: 60.4%, 96.6%) of patients were free from device- or procedure-related AEs at 30 days. Three procedure-related AEs were reported. One patient experienced right buttock pain approximately two weeks after subsequent EVAR, deemed by the investigator as related to the embolization procedure. Two patients experienced gastrointestinal disorders deemed possibly related to the procedure by the investigator; one was an SAE requiring additional hospitalization (possibly device- or procedure-related) and the second patient was seen by a vascular registrar and treated with paracetamol (acetaminophen). The severity of the three events was reported as mild, and the symptoms resolved without further intervention. Finally, 100% of investigators answered “yes” to the questions of whether the device was easily tracked and delivered to the target embolization site and was deployed accurately. The third question of whether the device was fluoroscopically visible during the procedure was answered “yes” by 96.4% of investigators. One investigator reported that the radiopaque markers on a Caterpillar 027 Micro device were difficult to visualize under fluoroscopy; the device, however, tracked easily and was delivered accurately to the embolization site without a reported AE.

## Discussion

Observations from this prospective, multicenter, first-in-human trial demonstrated that the Caterpillar embolization device could be deployed accurately to achieve arterial occlusion with few adverse events. All sites were successfully occluded during the embolization procedure, and only one potentially device-related SAE was reported through 30 days. A 69-year-old male received a Caterpillar 038 device to embolize the inferior mesenteric artery prior to EVAR; the site occluded within five minutes and the subsequent endovascular procedure was completed successfully. The patient complained of abdominal bloating and discolored stool 17 days post-embolization that required re-hospitalization, and the investigator determined that the SAE was possibly related to the embolization procedure or device. The incident was reported resolved at 35-days post-procedure without the need for further intervention.

The CHRYSALIS trial was designed to assess the Caterpillar embolization device in peripheral arteries ranging between 1.5- and 7-mm, vessel sizes that fit within device sizing parameters outlined in the instructions for use. Arteries treated included inferior and superior mesenteric, accessory renal, superior gluteal, common and internal iliac, and iliac artery side branches with a mean artery diameter of 4.5 mm (range 3–5 mm). Emergent cases with active bleeding were not specifically excluded, but active bleeding cases were not feasible due to patient screening requirements prior to study enrollment. All but one site was embolized in preparation for EVAR while the remaining site was treated post-EVAR because of a type II endoleak. EVAR cases provided the best chance of patient enrollment since the trial took place during the height of the COVID-19 pandemic. Cases could be planned well in advance, and therapeutic embolization of the internal iliac artery and side branches is a common clinical indication for embolic coils and plugs needed in approximately 20% of patients with aortic endografts and in about 80% of cases with common iliac artery aneurysms to prevent retrograde flow into the aneurysm sac [6, 10, 11]. Coils have been used for over twenty years with numerous retrospective reviews reporting on their use [10–13]. More recently, vascular embolization plugs have provided an alternative with similar results reported peri-procedure and through three months [6, 14–16]. On average, however, more coils are needed per patient compared to plugs and 30% of patients treated with coils needed additional embolization at the time of the endograft procedure [6].

There are obvious advantages to minimizing the number of embolization devices used per procedure including reduced procedure time, radiation exposure, and potentially overall cost. In the current study, most embolization sites were treated with one device (91.7%), an average of 1.1 devices per patient. Two sites required an additional Caterpillar device to achieve full occlusion. An accessory renal artery, 4 mm in diameter, was embolized with an 038 Caterpillar device; after eight minutes, the investigator decided to deploy a second 038 device which occluded the vessel within one minute. In a second case, a 6-mm right internal iliac artery was embolized with an 056 device; flow persisted at 19 min, so a second 056 device was deployed achieving occlusion within two minutes. Although the current work is not directly comparable to other devices, the use in most cases of one device to achieve occlusion is consistent with the literature on the use of the Amplatzer vascular plug for similar vessels and applications. Ryer and colleagues in a retrospective review of coils versus the Amplatzer plug reported a mean of 1.1 ± 0.4 plugs used compared to 5.8 ± 0.4 coils in internal iliac arteries prior to aortic endograft placement while Grenon et al. also reported a mean of 1.1 plugs used in addition to four patients requiring additional coil embolization [10, 16]. Khan et al. in their database review reported on average one plug compared to a mean of 7.6 coils, and Vandy et al. reported 1.4 plugs compared to 7.5 coils [7, 11]. Devices in all cases in this trial were sized correctly, and the need for additional devices in a few cases is consistent with the literature. The need for multiple devices may have to do with the vessel landing zone, anatomical variants, and vessel compliance but are beyond the scope of this study.

The average procedure duration in the CHRYSALIS trial was just under 12 min with a device deployment time about two minutes. Mean radiation exposure during the procedure was four minutes (median 2 min). Burbelko and colleagues reported a mean procedure time per vessel of 12 ± 8 min, a mean fluoroscopy time of 9.2 ± 4.5 min, and a mean deployment time of 6.2 ± 1.1 min using the Amplatzer plug to embolize similar vessels, and Warein et al. reported a mean fluoroscopy time of 14.2 ± 9.0 min [17, 18]. Although again retrospective and not directly comparable, procedural observations from the current study are similar to prior reports with other plugs. Vessel occlusion in the current trial was achieved in less than 10 min (91.6%) with well over half (62.5%) occluded within 3 min. Although two embolization sites took longer than ten minutes to occlude, all were occluded before the end of the procedure without the aid of adjunctive devices or procedures.

All adverse events in the trial were reported by the investigators. There was no core laboratory or clinical events committee to review and adjudicate adverse event reports; however, a medical monitor did assess any overall safety trends. Eleven patients reported at least one AE (24 events) through the course of the study with 84.2% of patients free from device- or procedure-related AEs at 30 days. One patient died. A 69-year-old male had a therapeutic embolization of the inferior mesenteric artery completed with an 056 Caterpillar device and the vessel occluded successfully within three minutes of the procedure. During a subsequent abdominal aortic endograft procedure the following day, the patient died from a rupture of his aortic aneurysm. The death was deemed by the investigator as unrelated to the embolization device or procedure.

Limitations of the CHYRYSALIS study should be noted. Although prospective and multicentered, it was designed as a first-in-human, feasibility study. The sample size was small, there were no hypothesis-tested endpoints, and all data were exploratory. Results were based on investigator visual inspections and judgment. Angiographic images were not reviewed by a core laboratory, and adverse events were not adjudicated by a clinical events committee. The Caterpillar embolization device tested in the current study may have performed differently than embolization devices and agents used in other trials. Differences between embolization devices and methods would require properly powered, randomized concurrently-controlled studies within similar vessels and demographic populations. Finally, the device was used prior to EVAR to prophylactically prevent Type II endoleaks in all but one patient. Devices were not used in emergent cases to control active hemorrhaging. Additional studies are needed to determine outcomes in other vessel beds and applications such as devascularization of neoplastic and non-neoplastic tissues as well as other types of aneurysms, pseudoaneurysms, or vascular malformations. Device sizes, number of devices used, and times to vessel occlusion may vary when the device is used in other applications.

In this first-in-human study, the Caterpillar embolization device was placed successfully to embolize peripheral arteries. The device provided 100% technical success with full occlusion typically with one device (91.7% of cases) generally achieved in less than 10 min. The CHRYSALIS trial also showed that the Caterpillar device could be used with minimal risk of a device- or procedure-related SAE (i.e., freedom from device- or procedure-related SAE of 94.7% at 30 days). Finally, investigators rated the device as accurate to place and easy to deliver with acceptable visibility under fluoroscopy, all necessary characteristics for successful embolization.

## Declarations

### Registration Clinicaltrials.gov

Unique Identifier NCT04090320.
